# Dietary supplementation of new-born foals with free nucleotides positively affects neonatal diarrhoea management

**DOI:** 10.1186/s13620-025-00294-3

**Published:** 2025-03-01

**Authors:** Livio Penazzi, Eleonora Pagliara, Tiziana Nervo, Ugo Ala, Andrea Bertuglia, Giovanna Romano, Jasmine Hattab, Pietro Giorgio Tiscar, Stefania Bergagna, Giulia Pagliasso, Sara Antoniazzi, Laura Cavallarin, Emanuela Valle, Liviana Prola

**Affiliations:** 1 Department of Veterinary Sciences, University of Turin, Largo Braccini 2, Grugliasco, 10095 Italy; 2 Centro Equino Arcadia, Frazione Mottura 106, Villafranca Piemonte, 10068 Italy; 3 Department of Veterinary Medicine, University of Teramo, SP18 Piano d‘Accio, Teramo, 64100 Italy; 4 Istituto Zooprofilattico Sperimentale Piemonte, Liguria E Valle d‘Aosta, Via Bologna 148, Turin, 10154 Italy; 5 Consiglio Nazionale Delle Ricerche, Largo Braccini 2, Grugliasco, 10095 Italy

**Keywords:** Nutrition, Microbiota, Volatile fatty acids, Equine, Neonatology, Supplement, Nutrient, Nucleotide

## Abstract

Foals commonly experience diarrhoea in the first weeks of life. Although this condition is rarely life-threatening, it can have significant health consequences. This study investigated whether new-born foals can benefit from a dietary supplement of nucleotides, as already demonstrated in other species. Dietary nucleotides have positive effects on rapidly proliferating tissues and are considered “semi-essential nutrients” since cells have only a limited capacity to synthesize these compounds. The aim of this study was to investigate whether providing foals with a dietary nucleotide supplementation, in the form of an oral paste, was able to affect diarrhoea incidence, systemic immunity, intestinal microbiota and volatile fatty acid production. Thirty new-born standardbred foals, from 3 different premises within the same area, were equally distributed between two groups: one group received an oral paste containing dietary nucleotides (NUCL group), while the other received a placebo paste (CTRL group). Faecal and blood samples were collected on days 1 and 35 after birth. No statistical differences in cytokines (TNF-α, IFN-γ, IL-6, IL-12) or faecal calprotectin levels were found between the two groups, suggesting that the level of nucleotide supplementation used in this study did not have significant effects on the systemic immune system and on the levels of faecal calprotectin. However, the NUCL group showed a lower relative frequency of number of days with diarrhoea (6.12% vs 13.33%; *p* < 0.001) and greater weight gain compared with the CTRL group (50.3 ± 5.65 kg vs 44.0 ± 8.65 kg; *p* < 0.05). Total volatile fatty acids, branched volatile fatty acids, acetic acid, propionic acid, butyric acid, succinic acid and iso-butyric acids in faecal samples were all higher in the NUCL group compared with the CTRL group. This outcome may explain an earlier establishment of a gut microbiota in the foals of the NUCL group that was closer to that typical of an adult horse, characterised by predominant fibrolytic populations. Volatile fatty acid production (especially butyric acid) has also been shown to correlate with the intestinal well-being of the horse, supporting the use of dietary nucleotide supplements for improved health and well-being in new-born foals. Although we noted no clear differences in the faecal microbial communities between the two groups, dietary nucleotide supplementation did appear to have a positive clinical outcome, reducing the number of days of diarrhoea and increasing the levels of volatile fatty acids.

**Supplementary Information:**

The online version contains supplementary material available at 10.1186/s13620-025-00294-3.

## Background

Diarrhoea in foals is common [1], with up to 85% of neonatal foals experiencing transient loose stools within the first weeks of life [2], especially between 5 and 15 days after birth. Although a broad range of aetiologies have been reported (e.g. viral, bacterial and parasitic) [3], episodes of early juvenile diarrhoea most commonly coincide with the mare’s first post-partum oestrus, and are therefore referred to as “foal-heat diarrhoea” [4].That said, analyses of the dam’s milk failed to reveal any causative agent able to induce diarrhoea [5], and orphan foals fed milk replacers were still found to exhibit diarrhoea episodes, ruling out the hormonal framework of the mare as the cause in these cases [3, 4, 6]. Instead, the normal development of the gastrointestinal microflora has been identified as the probable explanation for this kind of diarrhoea [5, 7–10]. Even mild episodes of diarrhoea can significantly impact the health of foals and by consequence have economic consequences for the breeders [11]. In fact, although nutrition-related behavioural problems are unlikely to be observed in the animals as they develop, the foals suffering longer periods of diarrhoea (independently from type and cause) and related complications may exhibit reduced weight and growth performances compared with foals that recover faster [9]. One hypothesis is that prolonged foal-heat diarrhoea may be caused by persistent microbiota instability [12]. Since the intestinal microbiota provides an important line of defence against pathogen colonization, its stability is highly relevant for tissue invasion prevention [13]. However, previous attempts to control changes in the microbiota for diarrhoea prevention and management in foals proved to be largely unsuccessful or even resulted in the exacerbation of diarrhoea episodes [9, 14–18]. This is probably due to the unique characteristics of the developing foal’s microbiota, characterised by lower microbial diversity compared with older animals and a markedly distinct population structure during the first two months of life [19–21]. Considering that manipulation of the foal microbiota is a difficult task at present due to our limited knowledge of the effects of the different bacterial populations during this life stage [11, 21], the need for a different approach to diarrhoea prevention presents itself.

Nucleotides are commonly found in foods of both animal and vegetable origin as nucleic acids or free nucleotides [22]. Dietary nucleotides appear to have a positive effect on the microbiota in animals, such as piglets and chicken [23], as well as on brain development due to their effects on the gut microbiota–gut–brain axis in early life [24]. Furthermore, nucleotides have been described to exhibit beneficial effects in human neonates; in particular, they can favourably influence tissue growth and repair, immune status and lipid metabolism [25]. Even though the mechanism of action is still unclear it appears nucleotides act in maintaining the optimal function and high turnover rate of various tissues as well as in promoting the development during period of rapid growth [23]. Thus, dietary nucleotides are deemed to be positive for rapidly proliferating tissues, such as the intestine and the immune system, especially in situations where the tissues are unable to have their nucleotide needs met by de novo synthesis [26], the reason for which they are described as “semi-essential nutrients” [27].

This project investigated the effects of a dietary free nucleotide supplement on the gastrointestinal and immune health of new-born foals. The specific aims were threefold. The aim first was to evaluate the effects of the treatment on the [frequency of] diarrhoea episodes during the first weeks of a foal’s life and whether it was able to promote favourable foal development and growth, as previously demonstrated in other species. The second aim was to identify any changes in the faecal microbiota, and the third was to evaluate whether the treatment led to a shift towards a cellular-mediated immune response, which can be beneficial for the development of local gastrointestinal immunity.

The novelty of the project resides in the different approach (compared to previous studies) in which the nucleotide supplementation aims to modulate, rather than directly change, the gastrointestinal health and microbiota of the foal. Therefore, our hypothesis was that the supplementation could affect positively the foals by different aspects: increased growth rate, reduction of the incidence of diarrhoea episodes, promotion of cellular mediated immunity, and earlier shift towards a gut microbiota and fermentation by-products typical of an adult horse.

## Materials and methods

### Animals

Thirty standardbred foals were enrolled in a comparative study carried out over a single foaling season (February–May). The foals were selected from three different breeding centres (10 foals each) located within the same area (< 20 km radius). The management regime during the study was the same in all three breeding centres. All mares received their booster vaccination for tetanus and equine influenza in the last month of gestation and were dewormed with Ivermectin within 24 h after foaling. Mares were kept in the foaling boxes from 48 h prior to the expected time of birth. All foals received tetanus antitoxin within 24 h of birth. The foals remained in the foaling box for the first 48 h after birth, but were then accompanied to overgrazed paddocks during the daytime and returned to their respective boxes in the evening for the remainder of the trial.

### Study design

The study was approved by the Ethical Committee of the University of Turin (Ethical approval prot. n. 567, University of Turin).

The foals were enrolled in a longitudinal, double-blinded, randomized controlled trial and randomly allocated to two different groups: the treatment group (NUCL), which received an oral paste composed of nucleotides, molasses and maltodextrins; and the placebo group (CTRL), which received an oral paste composed of molasses and maltodextrins alone. The study design. For each breeding centre foals were allocated and equally divided to each breeding centre following a cluster randomised allocation so that 5 foals received the placebo and 5 foals received the nucleotides in each setting. The paste was administered once a day, between 07:00 and 09:00 am, from day 1 after birth up until day 35 of the foal’s life. The oral paste of both groups had a uniform brown colour and was univocally identified for each foal rendering the operator blind to each foal’s treatment. In the treated animals the quantity of nucleotides administered during the study period was calculated as 0.2%/kg of dry matter (DM) of ingested milk. The level of milk ingestion (% of DM based on foal’s body weight) was derived from a previous study, according to Oftedal et al. [28]. As the foal total DM ingestion was growing, due to the increase in BW, the daily dosage of nucleotides was increased from 3.5 g up to 5 g at the end of the trial in order to maintain the level of inclusion as stated above.

### Inclusion criteria

Inclusion criteria at the birth were: APGAR score ≥ 9/10 [29–32], with respiratory rate, heart rate and rectal temperature within normal ranges; no navel or joint effusions; bright and alert mentation; and IgG ≥ 800 mg/dL, assessed using to a commercial semi-quantitative blood test (SNAP Foal IgG Test IDEXX®). During the trial the foals were clinically checked daily and any symptoms other than diarrhoea were considered exclusion criteria for that foal. None of the foals experienced more than 5 days of moderate or severe diarrhoea, using the modified faecal score from John et al. [9] described in Table 1. However, had a foal experienced moderate to severe diarrhoea (faecal score of 2–3/3) for more than 5 days the management protocol in place foresaw the administration of a commercial supplement based on diosmectite (ACME Smigol®) to reduce the severity of the episodes. Similarly, had moderate to severe diarrhoea (faecal score of 2–3/3) persisted for more than 7 days, the foals would have been excluded from the trial and further diagnostic procedures carried out in an attempt to identify the cause of the diarrhoea.

**Table 1:** Faecal score modified from John et al. (2015). Score from 1 to 3 can be considered from mild to severe diarrhoea

Score	Faecal quality	Severity of diarrhoea
0	Well-formed faeces	No diarrhoea
1	Soft but still formed faeces	Mild diarrhoea
2	Soft/liquid faeces, not formed, without soiling of the hindquarters and/or tail	Moderate diarrhoea
3	Liquid faeces with soiling of the hindquarters and/or tail	Severe diarrhoea

### Sample collection and analysis

**Fig. 1:**
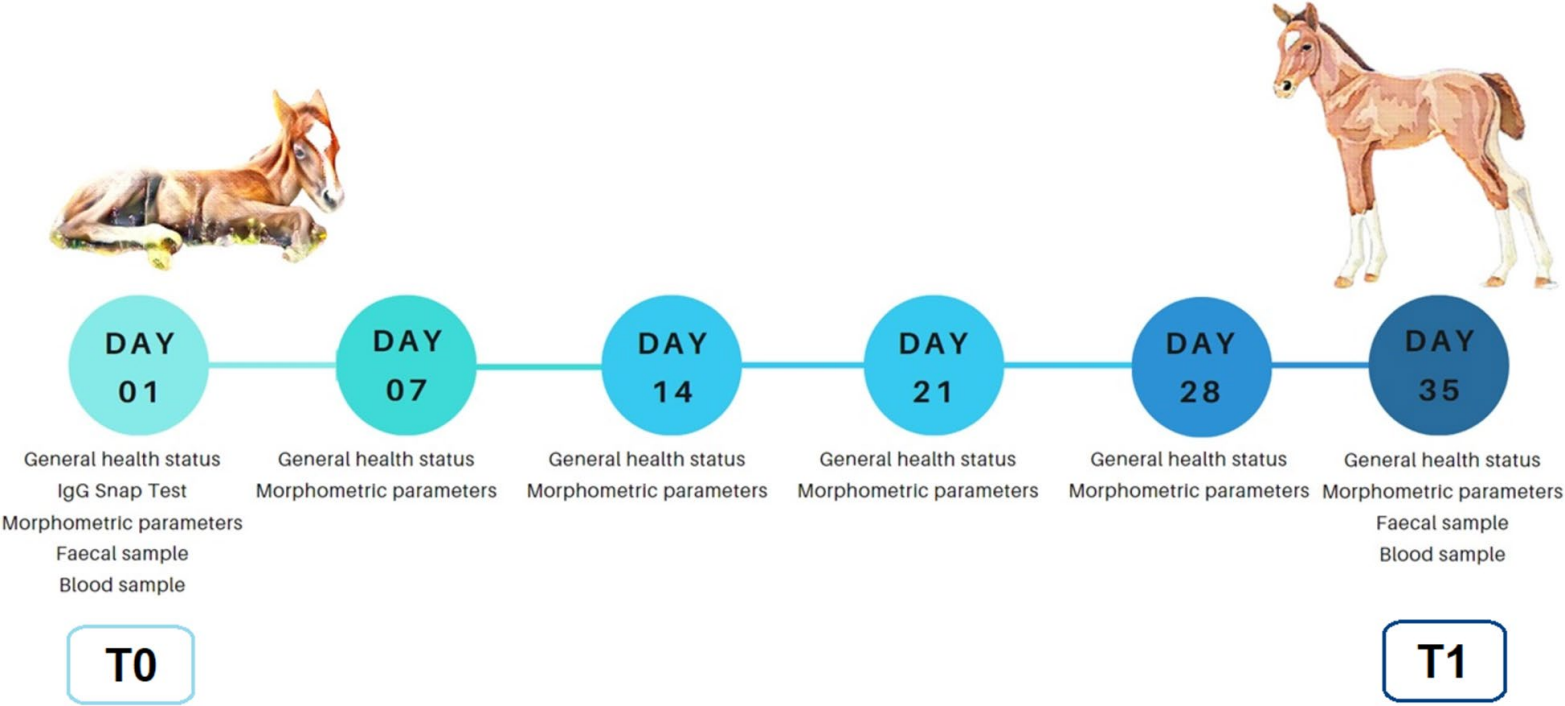
Timeline of the experimental procedures (image created using https://www.canva.com)

A timeline of the experimental procedures and sample collections, at day 1 (T0) and day 35 (T1) from foal birth, is summarized in Fig. 1.

Blood samples were collected from all the foals on days 1 and 35 after birth, and immediately centrifuged. Two aliquots of serum were stored at −80°C: one was used for electrophoresis (albumin, α1 e α2 -globulin, β1 e β2 -globulin and γ-globulin) and total protein analyses; the other was used for cytokine evaluation (TNF-α; IFN-γ, IL-6; IL-12). For the detection of total proteins, the stored serum samples were processed using the BT35500 automatic analyzer (Futurlab®, Limena, Italy) following the manufacturer's instructions. The reference values ​​are those set by the analyzer for equines (5.4–7.5 g/dL). For the electrophoresis analysis, we used a semi-automatic multiparametric biochemical analyzer (HYDRASYS LC SEBIA®, kit HYGRAGEL PROTEIN 15/30 and densitometer). We performed four immunoenzymatic tests, according to the indications and standard procedures provided by the manufacturer, for the detection of the following cytokines: TNF-α (Horse Tumor Necrosis Factor α BT-LAB Kit, Bioassay Technology Laboratory Shanghai, China); INF-γ (Horse Interferon γ BT-LAB Kit, Bioassay Technology Laboratory Shanghai, China); IL-12 (Horse Interleukin 12 ELISA Kit, MyBioSource, San Diego, CA); and IL-6 (Horse Interleukin 6 ELISA Kit, Bioassay Technology Laboratory Shanghai, China).

Samples of the first faeces produced within 48 h of the foal’s birth (i.e. first faeces after meconium) were collected from the ground immediately after the foal had defecated (within 15 min). The portion of faeces used for analysis was taken from the centre of the sample so as to avoid any contamination from the environment and then stored at −80°C for subsequent microbiota analysis. On day 35 a second faecal sample for microbiota analysis was collected using the same collection and storage methods; this time, a second faecal aliquot was stored at −20°C for the analysis of faecal calprotectin and volatile fatty acids. The faecal microbiota was analysed through 16S rRNA gene sequencing. In brief, total genomic DNA was extracted under a laminar flow cabinet using a commercial kit for DNA isolation (E.Z.N.A® Universal Pathogen, Omega Bio-Tek, Norcross, GA, USA), according to the manufacturer’s instructions, and stored at −20°C until use. DNA quality was assessed for each sample by Qubit fluorometer (Invitrogen). Extracted DNA samples were then normalized at 10 ng/µl. The microbiota was analysed through the sequencing of the V3-V4 region of 16S rRNA, which was amplified using the following primers: F, 5′- CCTACGGGNGGCWGCAG −3′, and R, 5′- GACTACHVGGGTATCTAATCC −3′. Primers were modified with forward and reverse overhangs (Forward overhang: 5’ TCGTCGGCAGCGTCAGATGTGTATAAGAGACAG; Reverse overhang: 5’ GTCTCGTGGGCTCGGAGATGTGTATAAGAGACAG‐[locus specific sequence]) necessary for dual index library preparation. Sequencing was performed on an Illumina MiSeq using a 2X300 flowcell for v3 chemistry. Data analysis was performed in QIIME 2 2021.11 [33]. Raw sequence demultiplexing and quality assessment was carried out using a q2 demux plugin. Denoising was performed by DADA2 via q2‐dada2) [34]. The amplicon sequences variants (ASVs) obtained were aligned by mafft via q2-alignement, and aligned sequences were then used to produce an approximately-maximum-likelihood phylogenetic tree using FastTree2 via q2‐phylogeny [35, 36]. Silva v138.1 was used as a reference for taxonomic annotation of ASVs [37, 38]. Read classification had a precision of 0.96 at the genus level, a recall of 0.93 and a F-measure of 0.95.

Faecal samples at T1 were also collected to assess faecal calprotectin and volatile fatty acids (VFA). Faecal calprotectin was assessed using a commercial ELISA kit (Horse Calprotectin ELISA Kit, MyBioSource, San Diego, CA). The analysis was conducted following the instruction of the kit after diluting the faeces in PBS (phosphate buffered saline) and collecting the supernatant after centrifugation at 3000 rpm for 20 min. Both Intra-assay CV (with CV (%) = SD/mean × 100) and Inter-assay CV (%) of the kit used were less than 15%. VFA quantification was carried out according to the methods described in Guantario et al. [39]. Briefly, samples were suspended at a ratio of 1:5 in 0.1 N H_2_SO_4_ solution, homogenized in a stomacher (Lab-Blender 400, Seward, Worthing, UK) for 5 min and centrifuged twice at 15,000 X g for 10 min at 4°C. The resulting extracts were filtered through filter paper and then through a 0.22 μm pore syringe filter. High performance liquid chromatography (HPLC) was conducted using a Dionex Ultimate 3000 (Thermo Fisher) with autosampler equipped with a 300 × 7.8 mm Aminex HPX-87H (Bio-Rad) and a guard-column. Injected samples (30 μL) were isocratically separated in 0.005 N H_2_SO_4_ at a flow rate of 0.6 mL/min at 41°C. VFAs were detected by UV light at 210 nm and identified using an external standard curve.

On day 1, and then every 7 days, each foal was weighed on a previously calibrated scale provided to each of the breeding centres. Morphometric parameters (girth circumference, withers to sacrum length and forelimb cannon bone circumferences) were assessed to evaluate foal growth. To assess the quality of each foal’s faeces on day 1 through to day 35, we used a modified (and simplified) version of the faecal scoring scale published by John et al*.* [9] (Table 1). In each facility the faeces were examined daily when the mares and foals were to their respective boxes for the night time.

### Statistical analysis

Statistical analysis and figure production were performed in R v4.1 [40].

Cytokine levels, faecal calprotectin and relative frequency of number of days of diarrhoea were compared between groups (CTRL vs. NUCL) and breeding centres using two-way ANOVA, considering the diet group and the breeding centre as the sources of variation. Before testing for differences between the two treatment groups and three breeding centres, the normality of the data distribution and the homogeneity of variance were assessed by the means of the Shapiro–Wilk test and Levene test, respectively. A Student’s t-test was used to evaluate weight gain (T1-T0) after checking for normality and homoscedasticity using the abovementioned tests. The significance level was set at *P* < 0.05. A statistical trend was considered for 0.05 ≤ *P* < 0.10.

Chao1, Faith’s phylogenetic diversity, evenness, observed features, and Simpson and Shannon indexes were used to evaluate Alpha diversity [41–45]. Alpha diversity differences among groups, breeding centres and time were evaluated using Kruskal–Wallis test. Beta diversity was estimated via q2-diversity by weighted UniFrac [46], unweighted UniFrac [47], Jaccard distance and Bray‐Curtis dissimilarity metrics [48, 49]. The permutational multivariate analysis of variance (PERMANOVA) test was used to evaluate differences in gut bacterial communities between groups based on 1000 permutations [50]. Results were considered statistically relevant for *P* < 0.05. Principal coordinate analysis (PCoA) was used to compare the different subjects. PCoA results were plotted in 3 dimensions, and ellipses highlighted microbiota clusters of foals divided by treatment group (Jaccard MetaMDS) with each dot representing the bacterial microbiota of a foal at T0 or T1.

A linear mixed-effects model (lme4 v. 1.1–35.3, run in R environment v. 4.4.0 and Rstudio [Build 524]) was used to determine a predictive relationship between body weight and morphometric parameters (girth circumference, withers to sacrum length and forelimb cannon bone circumferences), dietary regimen and a possible interaction between the two, regarded as fixed-effects. Individual subjects were treated as random effects. The conditional coefficient of determination (conditional R^2^), for both the fixed and random effects, and marginal coefficient of determination (marginal R^2^) for the fixed effects only, were evaluated using the sjstats package (v. 0.19.0).

## Results

A total of 30 foals were enrolled onto the study. One foal was subsequently removed from the study for reasons not related to the trial itself (the mare and foal were transferred to another breeding centre one week after the start of oral paste administration). None of the foals experienced more than 5 days of moderate to severe diarrhoea, meaning that all 29 foals (15 in the CTRL group and 14 in the NUCL group) completed the trial and were included in the statistical analysis.

At both T0 and T1 blood serum electrophoresis parameters were within the normal reference ranges according to Bauer et al*.* [51]. There were no statistical differences in the levels of cytokines (TNF-α; IFN-γ, IL-6; IL-12) or faecal calprotectin between treatment groups or according to the breeding centre variable at either T0 or T1 (please see Table S1 and S2 in the supplementary materials).

**Fig. 2:**
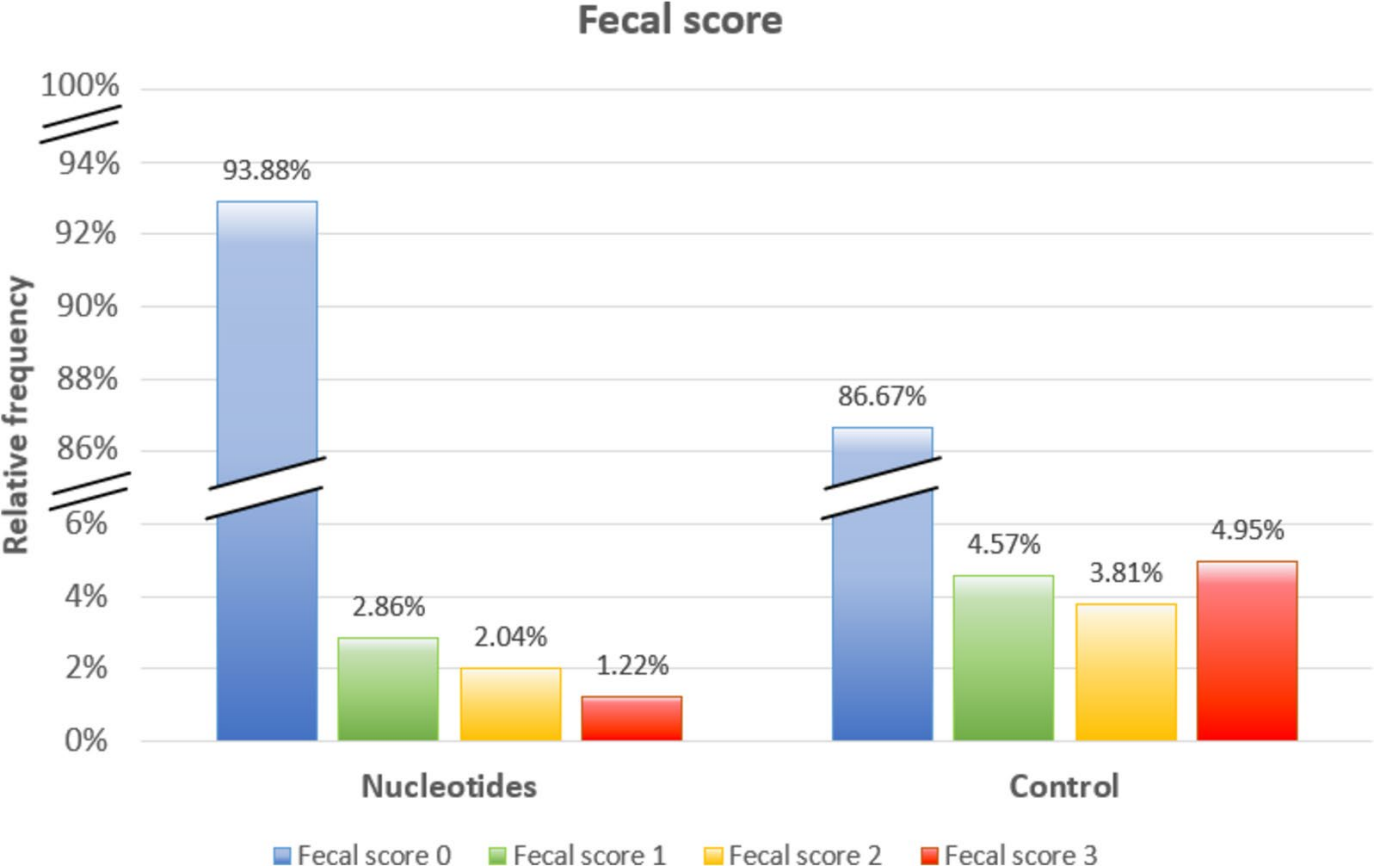
Faecal score relative frequency score (during the days with diarrhoea) in the NUCL and CTRL groups

The relative frequency of days with diarrhoea was significantly higher (*p* < 0.001) in the CTRL group (13.33%, 70/525 days) than in the NUCL group (6.12%, 30/490 days). Furthermore, the faecal score relative frequency (during the days with diarrhoea) was less severe in the NUCL group compared to the CTRL group as shown in Fig. 2.

**Fig. 3:**
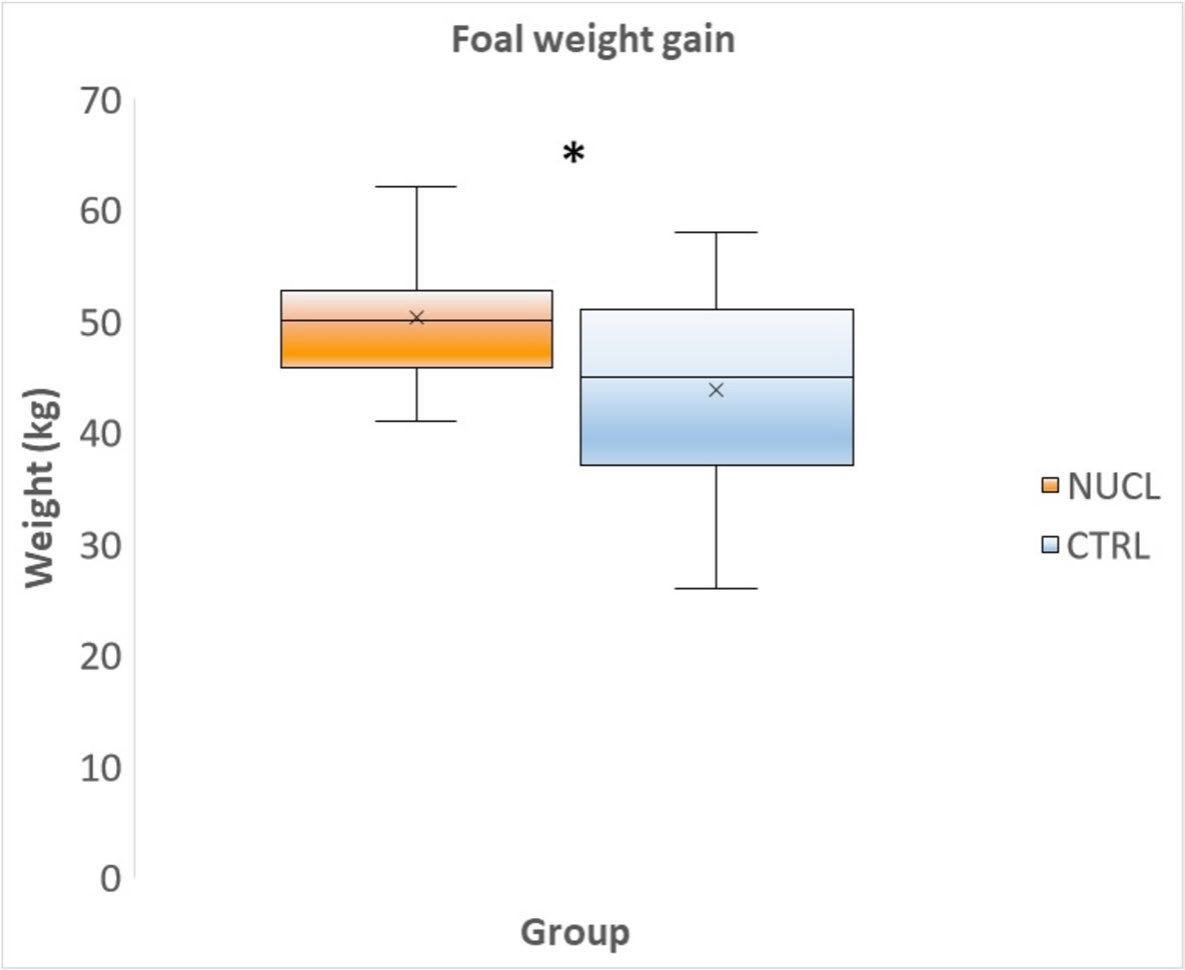
Foal weight gain (kg) at the end of the study (T1-T0) in the NUCL group compared to the CTRL group (*p < 0.05)

**Fig. 4:**
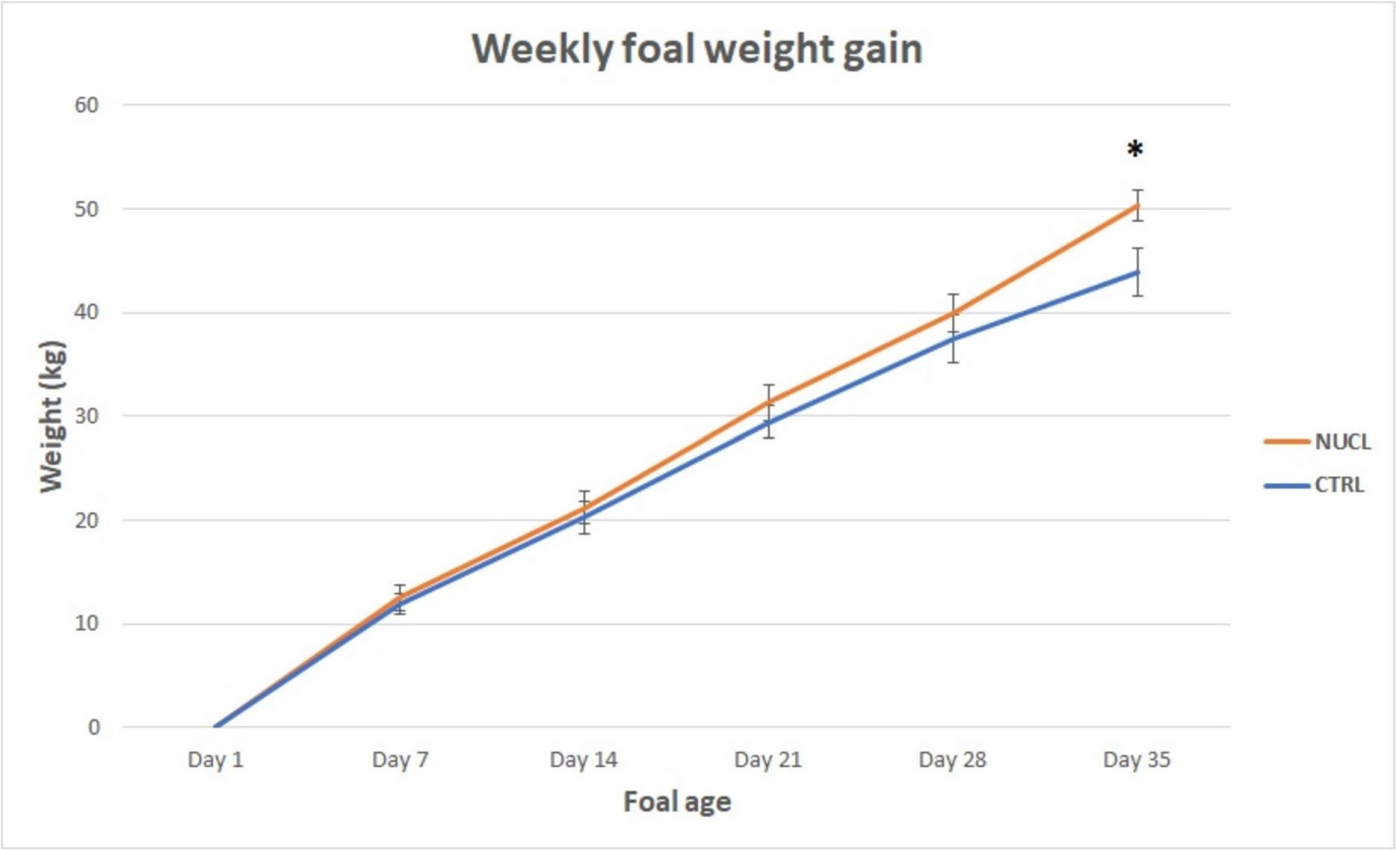
Weekly foal weight gain (kg) in the NUCL group compared to the CTRL group (values as mean ± SEM) starting from T0. *p < 0.05

**Fig. 5:**
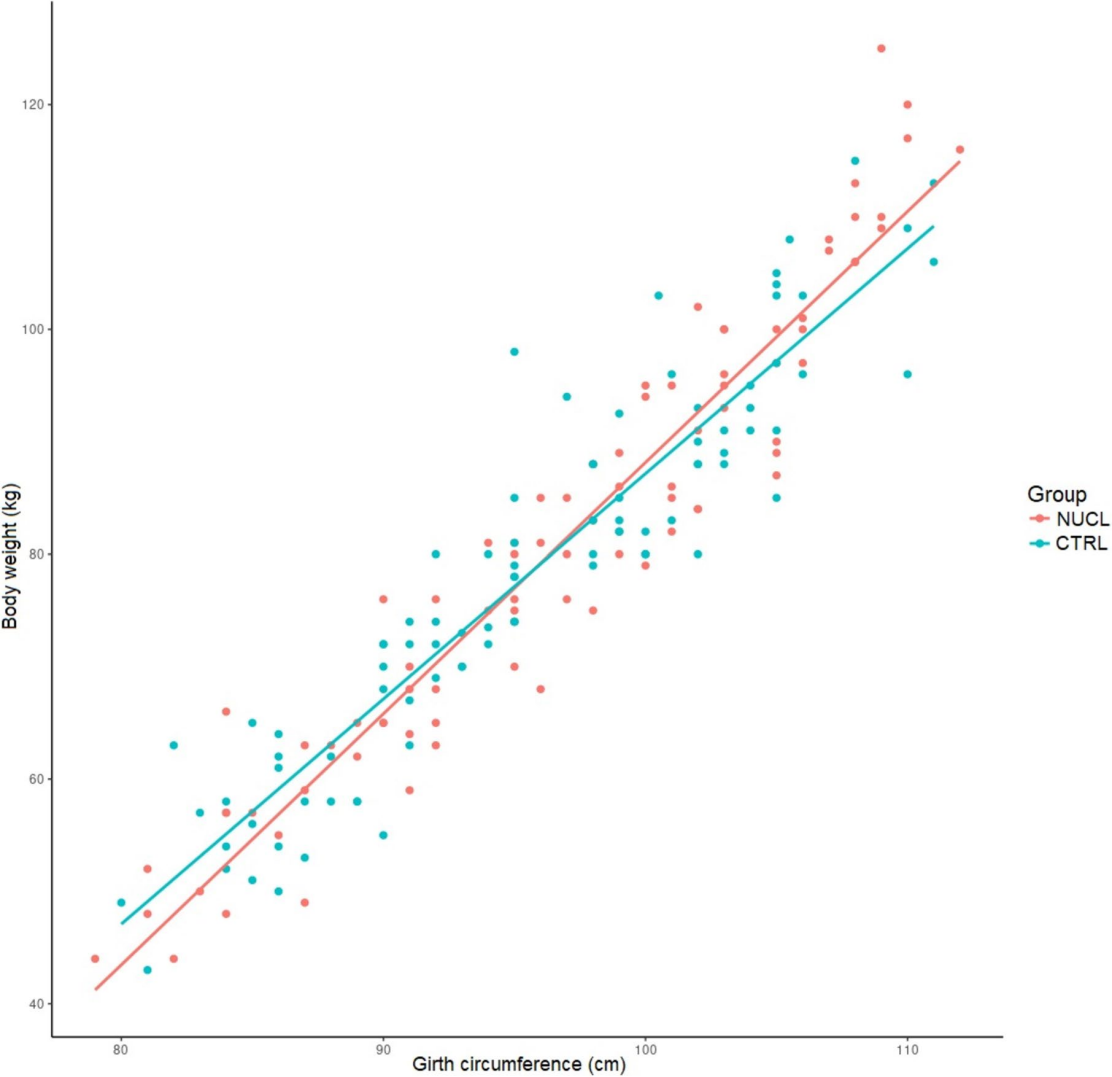
Relationship between girth circumference (GC, cm) and body weight (BW, kg) in the NUCL (BW = −136.5 + 2.24 GC; *n* = 14) and CTRL group (BW = −114.3 + 2.01 GC; *n* = 15). The conditional coefficient of determination, conditional R^2^, for the model was 0.923 and the marginal coefficient of determination, marginal R.^2^, was 0.891

The foals in the NUCL group exhibited greater (*p* < 0.05) weight gain (50,3 ± 5,65 kg) compared with the CTRL group (44 ± 8,65 kg), as well as a faster growth rate (Figs. 3 and 4). In addition, of the morphometric parameters evaluated, the girth circumference (GC) was the most reliable for estimating foal body weight (BW) from day 1 up to day 35 of life (Fig. 5).

Table 2 reports the results of the VFA analysis conducted on faecal samples obtained at T1, and any interactions between diet and breeding centre (diet*breeding centre). The foals of the NUCL group showed significantly higher faecal concentrations of total VFAs, branched VFAs, acetic acid, propionic acid, butyric acid, succinic acid and iso-butyric acid compared with the foals in the CTRL group. No effect of breeding centre or diet*breeding centre interaction was detected.

**Table 2:** Total faecal and individual VFA concentrations (mg/100 ml) at T1 in foals from NUCL and CTRL groups. P values are for dietary group and breeding centre as possible sources of variation, and for the group*breeding centre interaction

Analytes	Group			P value		
VFAs	NUCL	CTRL	SEM	Breeding centre	Group	Group*breeding centre interaction
Total VFAs	153.37	93.78	9.54	0.60	0.0016**	0.72
Branched VFAs	70.33	46.42	5.05	0.98	0.023*	0.75
Acetic acid	55.78	31.77	4.33	0.58	0.0046**	0.41
Propionic acid	15.98	8.70	1.15	0.56	0.0009***	0.44
Butyric acid	7.78	4.44	0.73	0.62	0.026*	0.79
Succinic acid	11.83	5.66	0.98	0.63	0.0011**	0.42
Valerianic acid	3.52	2.44	0.52	0.23	0.31	0.66
Iso-butyric acid	65.58	40.55	5.00	0.98	0.016*	0.86
Iso-valeric acid	4.74	5.87	0.87	0.13	0.51	0.49

*VFA *Volatile fatty acids, *SEM *Standard error of the mean, *NUCL *Nucleotide group, *CTRL *Control group. statistical significance: *P < 0.05; **P < 0.01; ***P < 0.001

**Fig. 6:**
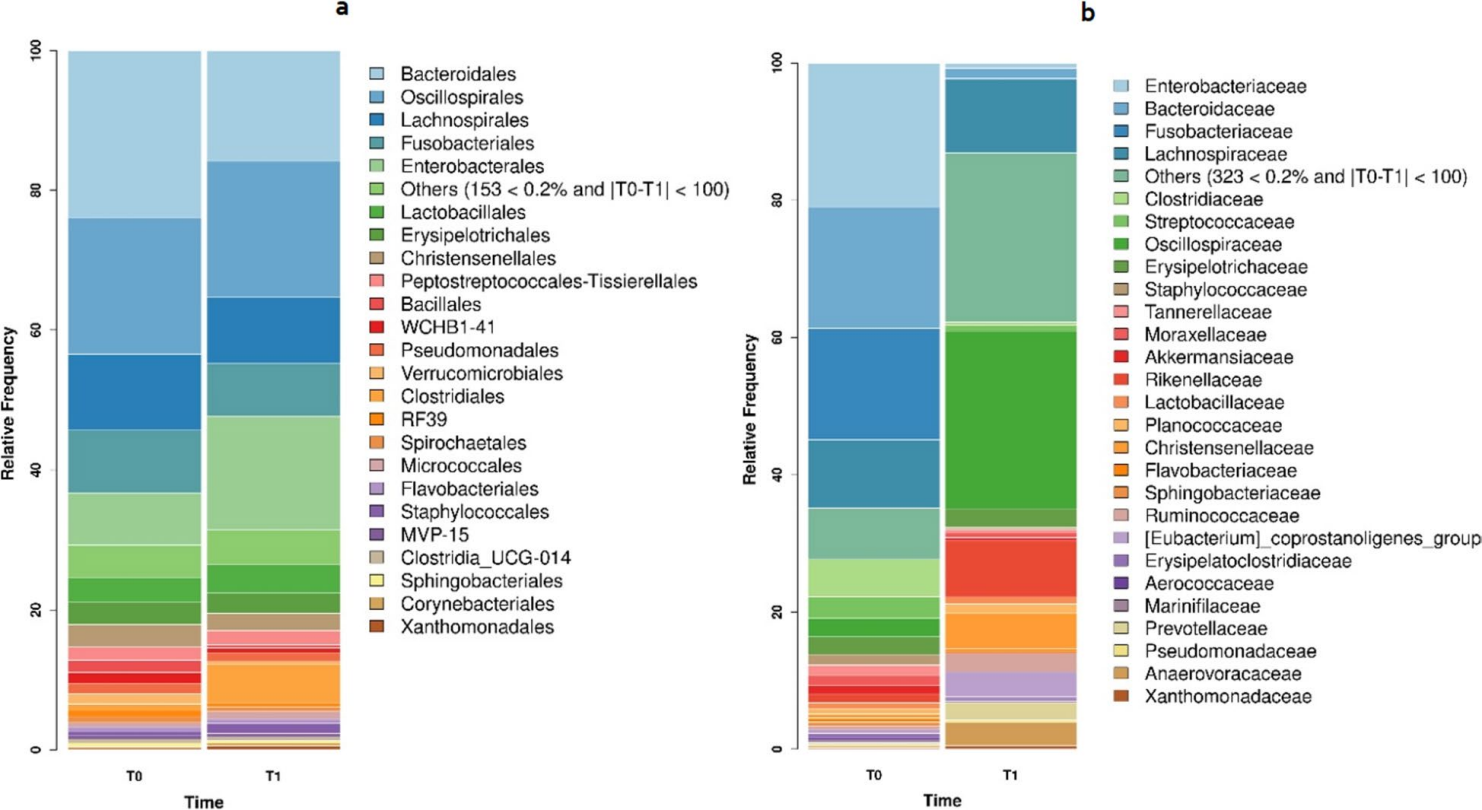
Average faecal bacterial microbiota of the foals at T0 (NUCL and CTRL clustered) and T1 (NUCL and CTRL clustered). Bacterial microbiota structures at the order (a) and family (b) level on day 1 (T0) and day 35 (T1) of birth. Only orders and families accounting for a relative abundance > 1% of the total reads are listed individually; the remainder are clustered into “others”. The two experimental groups shared the same predominant orders and families, but a change in the relative abundance of each order was observed at T1 with respect to T0

For microbiota analysis, a total of 54 samples were included in the final analysis, yielding to the identification of 5,822 features and a total feature frequency of 2,085,017. The number of reads per sample ranged from a minimum of 1,576 to a maximum of 74,164, with single read lengths ranging from 210 to 235. Overall, we identified a total of 180 orders, with Bacteroidales, Oscillospirales, Enterobacterales, Lachnospirales, Fusobacterales and Lactobacillales being the most abundant. The families with the highest relative abundances were: Oscillospiraceae, Enterobacteriaceae, Lachnospiraceae, Bacteroidaceae, Fusobacteriaceae and Rickenellaceae (Fig. 6). At T0, the orders with the highest relative abundances were: Bacteroidales (23.97%), Oscillospirales (19.46%), Lachnospirales (10.86%), Fusobacteriales (8.94%) and Enterobacterales (7.47%). At T1, the most represented orders remained unchanged, with Oscillospirales (19.42%) becoming the most abundant, followed by Enterobacterales (16.27%), Bacteroidales (15.82%), Lachnospirales (9.57%) and Fusobacteriales (7.49%). At the family level, Enterobacteriaceae (21.05%), Bacteroidaceae (17.75%), Fusobacteriaceae (16.21%), Lachnospiraceae (9.90%) and Clostridiaceae (5.47%) were the most abundant in T0, wheras Oscillospiraceae (25.87%), Lachnospiraceae (10.61%), Rickenellaceae (8.19%), Christensenellaceae (5.21%) and [Eubacterium]_coprostanoligenes_ group (3.61%) were the most prevalent at T1 (Fig. 6).

**Fig. 7:**
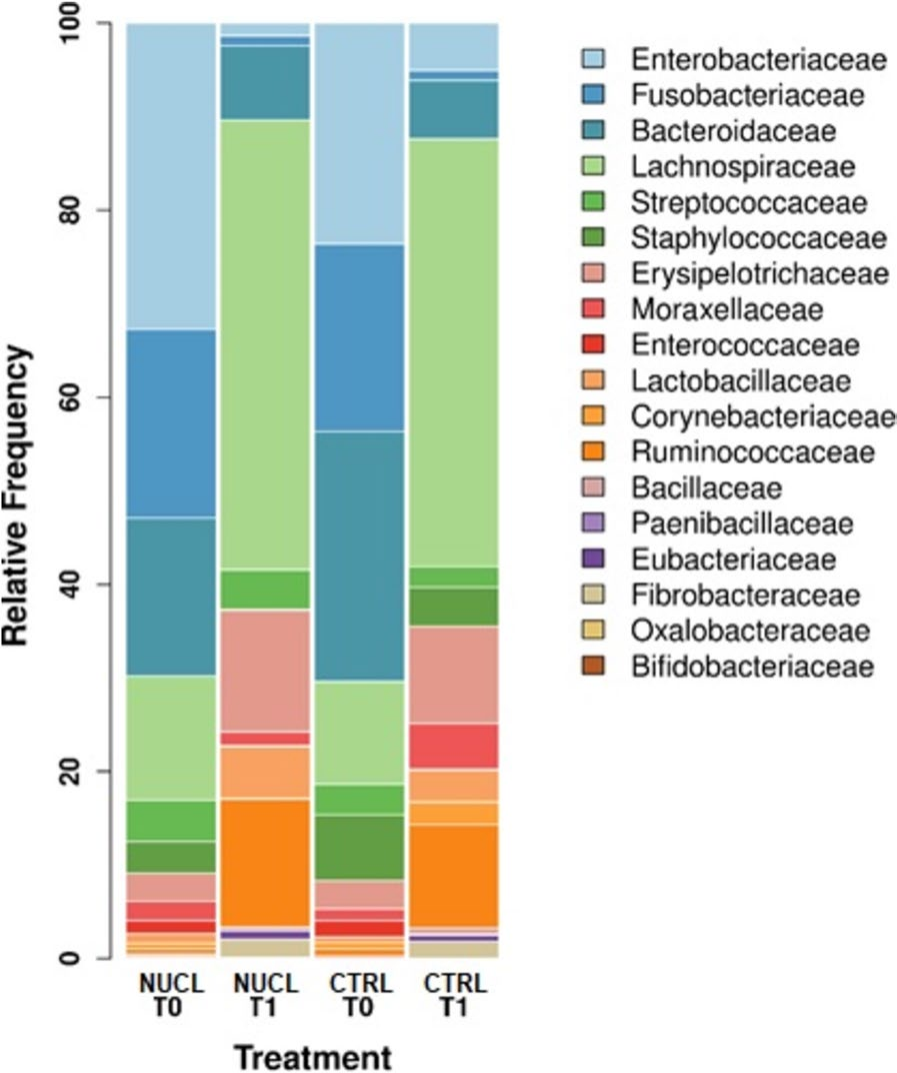
Average faecal bacterial microbiota of the foals divided according to time of collection (T0 vs T1) and treatment group (NUCL vs CTRL). Bacterial microbiota structures at the family level on day 1 (T0) and day 35 (T1) of birth. The two experimental groups share the same predominant families, but a change in the relative abundance of each family was observed between the two clusters

When comparing the samples according to the group, a statistically significant difference was revealed between groups at T1 (PERMANOVA, UnWeighted Unifrac *p* = 0.03). On the other hand, when comparing the samples according to sampling time (T0 vs T1), we observed a shift towards greater phylogenetic diversity (PERMANOVA, UnWeighted Unifrac *p* = 0.0001) and a significant degree of composition dissimilarity (PERMANOVA, Jaccard *p* = 0001) (Fig. 7).

**Fig. 8:**
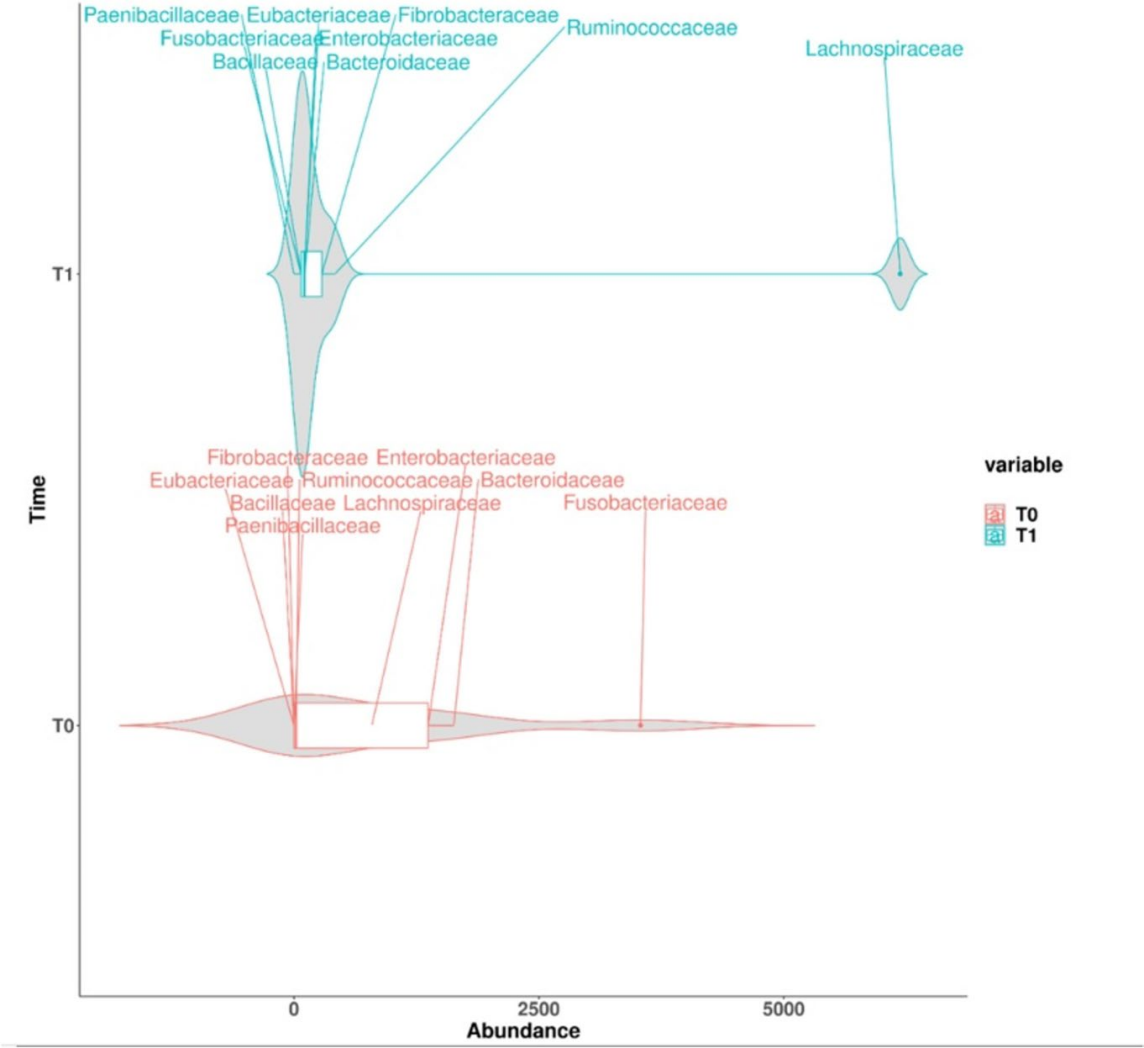
Violin plot showing the shift in the relative faecal abundances of bacterial families involved in digestion and volatile fatty acid production in foals between the day 1 (T0) and day 35 (T1) of birth. At T0 the examined families display a wider distribution pattern and are more abundant compared with at T1, when the abundance of each family was uniformly low, with the exception of Lachnospiraceae

The variation in the relative abundances of bacterial families involved in fibre degradation was investigated to assess for changes associated with foal feeding habits (i.e., start of hay and feed ingestion). A reduction in lactic bacteria was observed at T1, while the relative abundance of fibrolityc bacteria, such as the Lachnospiraceae and Ruminococcaceae families, increased (Fig. 8).

**Fig. 9:**
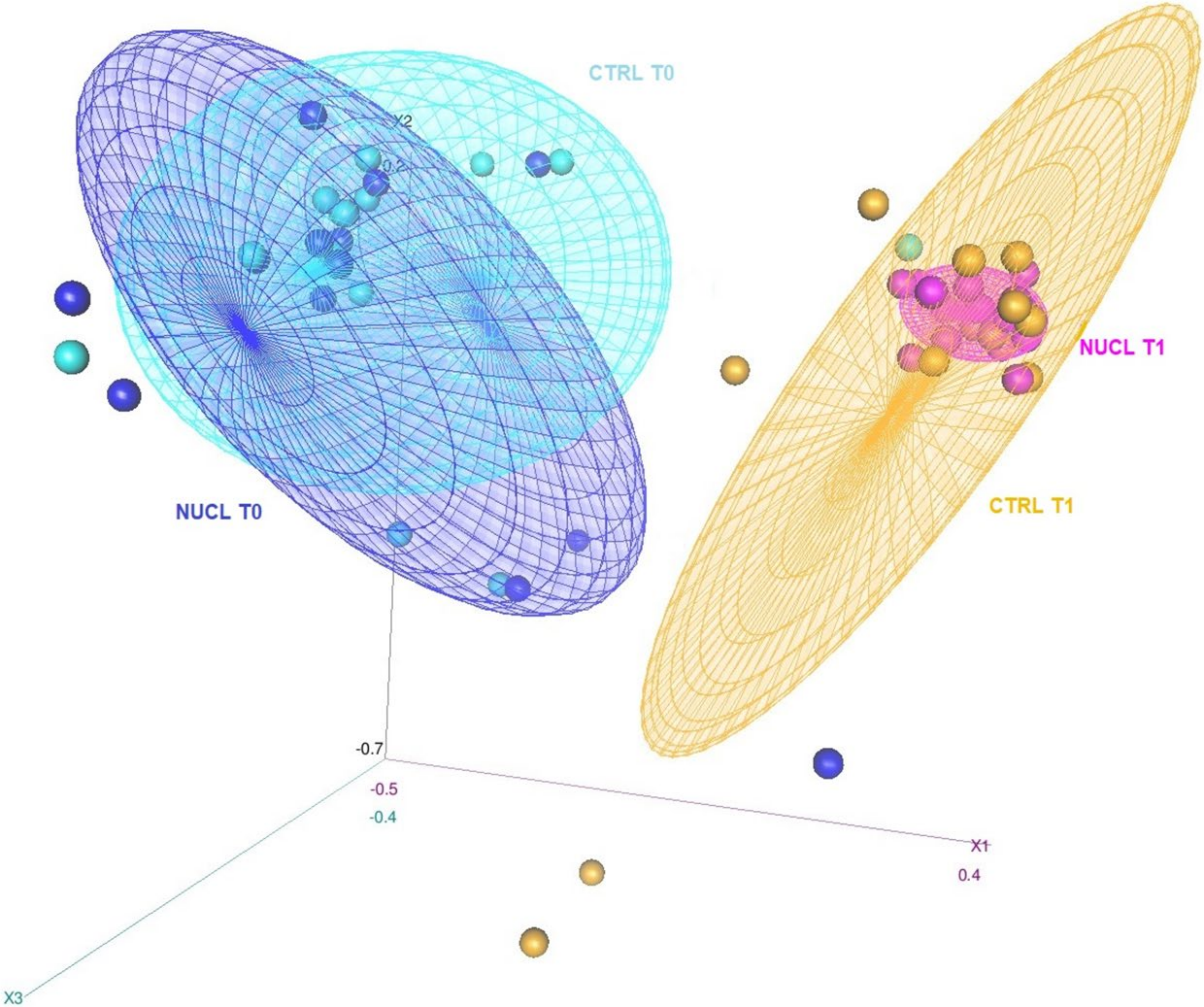
3D PCoA plot with ellipse showing microbiota clusters of foals divided according to treatment group (Jaccard MetaMDS). Each dot represents the bacterial microbiota of a foal at T0 or T1. The NUCL T0 and CTRL T0 clusters are distinct to those for NUCL T1 and CTRL T1, and NUCL T1 samples are more closely clustered compared with those for CTRL T1

The 3D PCoA plot shows the microbiota clusters divided according to treatment group and sampling time. While there is a clear clustering difference between T0 and T1 in both treatment groups, it can be noticed that the samples of the NUCL group at T1 gather together more closely compared with those of the CTRL group at T1 (Fig. 9).

## Discussion

To the best of our knowledge, the present field study was the first of its kind to explore the possible effects of dietary nucleotide supplementation in neonatal foals. Previous studies have attempted to ameliorate neonatal diarrhoea in foals through the use of both prebiotics and probiotics, but the reported findings are inconsistent, as outlined in a previous review [11]. Moreover, in addition to their poor efficacy, concerns (such as anorexia and colic) have been raised about the use of probiotics in foals [14, 17, 19, 52, 53]. Nonetheless, the impact of probiotics on the foal microbiota is still not well understood, and one possible explanation for the lack of efficacy as well as adverse enteric effects could reside in the differences between the adult horse enteric immune system and microbiota and those of a foal, in particular during their first 30 days of life [54]. Furthermore, human neonates have previously been described to benefit from dietary nucleotides [55, 56], thought to arise from the fact that intestinal mucosa, leucocytes, enterocytes and lymphocytes have a limited capacity to perform de novo synthesis of these compounds that are essential for cell proliferation [57]. By consequence, these tissues must obtain nitrogenous bases through salvage pathways involving exogenous sources of nucleotides [58, 59], making their supply in the diet important [60]. In our study, we observed a significant reduction in the relative frequency of diarrhoea episodes in the group receiving the dietary nucleotides compared with the control group. These results sustain the hypothesis that dietary nucleotides may positively affect the capacity of the intestinal mucosa to regenerate, considering their known role in promoting the development of cellular ultrastructure in the gastrointestinal system [61]. This finding is of great importance for foals since certain conditions, such as rapid growth, immunosuppression or gut injury, may deplete the endogenous availability of nucleotides, making their exogenous supply ever the more them conditionally essential, especially for the gastrointestinal and the immune systems [27, 62, 63]. Regarding immune function, we did not detect any significant differences between the two groups, as cytokines and faecal calprotectin levels remained similar. Previous studies on mice reported dietary nucleotides to boost cellular and local immunity [64–71]; while an increase in both cellular [72] and humoral [55, 73, 74] immune responses were noted in human infants. Similarly, studies on dietary nucleotide supplementation in piglets reported improved growth performance and immune responses [75–77]. However, immune function was assessed in response to vaccination in these studies, while we did not challenge the foals with a vaccination, and this could have influenced the possible response to the supplementation. The duration of nucleotide supplementation has also been reported to influence the effects on immune cell functions and proliferation [78]. Hence, while the 5 week supplementation period of the present study was sufficient to produce detectable effects on the gastrointestinal tract, it may not have been long enough to impact the immune system. Moreover, the cytokine analysis performed may not have been sensitive enough to detect any local changes affecting the intestinal cellular response.

Previous studies in animals [77, 79] and human neonates [80, 81] showed increased weight gain when dietary nucleotides were added to the diet. Similarly, the foals receiving the nucleotide supplementation in the present study showed greater weight gain compared with those in the control group. As hypothesized by Jang et al*.* [79], this effect could be a consequence of reduced intestinal inflammation and improved intestinal villi structure and energy digestibility. Nonetheless, the observed effects on the microbiota could also have played a role in their better growth performance. The importance of the microbiota in foal development is crucial as it can be affected by different dynamics, including exposure to transient, non-colonizing organisms via the mare and the environment [54], adaptation to rapid and continuous changes in the diet [21], and periods of coprophagic behaviour [8]. Marked changes in the microbiota can have a direct effect on foal health throughout the first 2 months of life, after which the bacterial populations tend to stabilize [1, 19–21]. The results of PCoA in our study revealed a tighter clustering of the relative abundances of faecal bacterial families in the supplemented foals at T1, indicating a better stabilization of their microbiota compared with the control group. In accordance with De La Torre et al*.* [10], who found increased levels of *Enterobacteriaceae* in diarrheic foals at seven days of age with respect to healthy foals, the CTRL group at T1 showed a higher relative abundance of this family compared with the NUCL group. Furthermore, Schoster et al*.* [1] reported that foals exhibiting a higher prevalence of diarrhoea had lower abundances of *Lachnospiraceae* and *Ruminococcaceae*, as also observed in the CTRL group of our study compared with the supplemented foals at T1.

Nucleotides are reported to enhance butyrate-producing bacteria and genera, such as *Ruminococcus* and *Intestinomonas*, in infants [82]. When considering composition changes (in both groups) between T0 and T1, only the Ruminococcaceae family showed an increase in relative abundance, while Lachnospiraceae was the only fibre-degrading bacterial family to exhibit an increase in relative abundance at T1. This family is regarded a plant polysaccharide-degrader and producer of butyrate [83]. As regards the low abundances of the other fibre-degrading bacteria investigated (Fig. 8, violin plot), this might be explained by the fact that other fibre-degrading organisms (such as fungi and yeasts) play a major role in this pathway in horses. In fact, the degradation of plant fibre is a complex process relying on the combined actions of fungi, bacteria and archaea, and recent studies have highlighted the prominent role of yeasts and fungi in the fibrolytic process [84].

Overall, the faecal bacterial communities found in the present study were in line with the literature, which reports the intestinal microbiota of new-borns to be “non-specific”, only converging over time towards an adult-like one [24]. The differences between the NUCL and CTRL groups were not very remarkable, but this may be due to the much greater differences that characterize the microbiota of new-born versus 35-day-old foals. More precisely, the larger time-dependent changes in the microbiota could have somehow masked smaller differences in the composition of the microbial communities. There is also some evidence that dietary nucleotides may promote the early determination of gastrointestinal bacterial communities, supporting the stabilization of microbiota, as observed in the present study via PCoA clustering. Thus, the higher level of clustering observed in the NUCL group could depend on the maturation of the gut bacterial communities fostered by nucleotide treatment. Furthermore, administration of different quantities of nucleotides was shown to produce different degrees of variation in the bacterial microbiota and metabolites within the host [85]. Further studies would be useful to investigate the correlation between the quantity of nucleotide ingestion and the extent of changes in the microbiota and immune function, as nucleotides administration seem to affect both of them [24]. Similarly, it may be worth exploring if early stabilization of the gut flora could have an effect in the long term during the horse life.

In the present work, foals from NUCL and CTRL group were also observed to have different clinical outcomes with respect to diarrhoea presentation. This is particularly relevant considering that both groups came from the same breeding farms and the animals were exposed to identical environmental and management conditions. In addition, significant differences were detected in faecal VFA between the two groups at T1, with higher total VFA in the NUCL group compared with controls. Volatile fatty acids (in particular butyrate) appear to regulate the expression of genes involved in controlling the proliferation, differentiation and apoptosis of intestinal epithelial cells [86]. Furthermore, VFA are the main and favourite metabolic substrate of colonocytes, providing them with approximately 60–70% of the energy requirements necessary for proliferation and differentiation in the equine colonic mucosa [87]. We must also remember that although faecal VFA concentration may not represent the actual caecal levels of VFA, they can provide indirect information about changes in VFA production [88]. Julliand et al*.* [89] reported that the concentration of fibrolytic bacteria and acetate production were lower in horses fed a diet based on hay and barley compared with a diet of hay only. In our study we saw an increase in acetate production in the NUCL group, which may explain a better adaptation of foals to a fibrous diet compared with the CTRL group. In general, in the foals receiving the nucleotide supplementation we observed a higher concentration of the main VFAs normally produced by the adult horse (namely, acetic acid, propionic acid and butyric acid) [90] compared with the CTRL group. This difference may mean that bacterial colonization of the hindgut and subsequent VFA production had occurred earlier in foals receiving nucleotides compared with untreated foals. This may mean that the foals receiving the nucleotides were able to adapt more readily to the dietary changes associated with the start of weaning. Another aspect that should be considered is the concentrations of butyric acid in faeces of foals treated with nucleotides. In particular, the concentration of faecal butyrate was higher in the foals of the NUCL group compared with in those receiving the placebo. This can be considered a positive aspect as butyric acid is a final product of the microbial fermentation of fibre, and some studies have shown how it positively influences the health of the intestine, promoting the differentiation of colonocytes, exerting an anti-inflammatory effect and modulating oxidative stress [91]. Thus, the greater concentration of faecal butyrate in the foals of the NUCL group could be considered an index of intestinal well-being, in particular of enterocyte health, thus also explaining the decrease in the incidence of diarrhoea in these foals. Finally, the higher level of iso-butyric acid in the foals receiving the nucleotide supplement may also benefit their health, being involved in the degradation of the plant cell wall, promoting their digestion [92].

The main limitations in this study were due to the relatively small number of faecal samples for the microbiota analysis and the lack of assessment of the mares microbiota for comparison. In addition, the lack of a vaccination challenge and the short duration of the trial may have concealed longstanding effects of nucleotide supplementation.

## Conclusions

The management of diarrhoea in foals presents a difficult task for both owners and equine veterinarians. As such, it is important to find new approaches to prevent and minimize these events. The use of dietary nucleotides in this study successfully decreased the incidence and gravity of diarrhoea episodes in new-born foals, as already demonstrated in other species. In addition, the increased growth rate recorded for the supplemented group and the beneficial changes noted in the gut microbiota further support the validity of using dietary nucleotides in these animals.

## Supplementary Information

Supplementary Material 1

Supplementary Material 2

Supplementary Material 3

## Data Availability

The datasets used and/or analysed during the current study are available from the corresponding author on reasonable request.

## Abbreviations

BW: Body Weight
CTRL: Control group
DM: Dry matter
GC: Girth Circumference
HPLC: High Performance Liquid Chromatography
NUCL: Nucleotide group
PCoA: Principal Coordinate Analysis
SEM: Standard Error of the Mean
VFA: Volatile Fatty Acids
