# Supplementary material for: Dietary supplementation of new-born foals with free nucleotides positively affects neonatal diarrhoea management
Source: Ir Vet J. 2025 Mar 1;78:7. doi: 10.1186/s13620-025-00294-3 (PMC11871744; doi:10.1186/s13620-025-00294-3)
Supplement: Supplementary file 1 — Supplementary Material 1 [file 13620_2025_294_MOESM1_ESM.docx]

Supplementary material

**Table S1**

Blood serum electrophoresis parameters concentrations and cytokine levels at T0 in foals from NUCL and CTRL groups. P values are for dietary group and breeding centre as possible sources of variation, and for the group*breeding centre interaction.

| Analytes |  | Group | |  |  |  | P value | | |
| --- | --- | --- | --- | --- | --- | --- | --- | --- | --- |
|  |  | NUCL | CTRL |  | SEM |  | Breeding centre | Group | Group*breeding centre interaction |
| Total protein (g/dL) |  | 5.93 | 5.77 |  | 0.194 |  | 0.88 | 0.68 | 0.20 |
| Albumin (g/dL) |  | 2.86 | 2.97 |  | 0.048 |  | 0.91 | 0.26 | 0.44 |
| α1-globulin (g/dL) |  | 0.15 | 0.16 |  | 0.006 |  | 0.47 | 0.44 | 0.24 |
| α2-globulin (g/dL) |  | 0.38 | 0.35 |  | 0.021 |  | 0.22 | 0.37 | 0.50 |
| β1-globulin (g/dL) |  | 0.56 | 0.51 |  | 0.037 |  | 0.23 | 0.45 | 0.11 |
| β2-globulin (g/dL) |  | 0.66 | 0.58 |  | 0.045 |  | 0.36 | 0.39 | 0.79 |
| γ-globulin (g/dL) |  | 1.31 | 1.18 |  | 0.111 |  | 0.41 | 0.58 | 0.24 |
| TNF-α (ng/L) |  | 78.4 | 87.2 |  | 3.026 |  | 0.11 | 0.15 | 0.17 |
| IFN-γ (ng/L) |  | 61.0 | 69.5 |  | 3.642 |  | 0.97 | 0.27 | 0.28 |
| IL-6 (ng/L) |  | 5.44 | 5.89 |  | 0.492 |  | 0.95 | 0.67 | 0.20 |
| IL-12 (pg/mL) |  | 49.1 | 37.3 |  | 5.538 |  | 0.84 | 0.30 | 0.93 |
