# Supplementary material for: Dietary supplementation of new-born foals with free nucleotides positively affects neonatal diarrhoea management
Source: Ir Vet J. 2025 Mar 1;78:7. doi: 10.1186/s13620-025-00294-3 (PMC11871744; doi:10.1186/s13620-025-00294-3)
Supplement: Supplementary file 2 — Supplementary Material 2 [file 13620_2025_294_MOESM2_ESM.docx]

Supplementary material

**Table S2**

Blood serum electrophoresis parameters concentrations, cytokine and calprotectin levels at T1 in foals from NUCL and CTRL groups. P values are for dietary group and breeding centre as possible sources of variation, and for the group*breeding centre interaction.

| Analytes |  | Group | |  |  |  | P value | | |
| --- | --- | --- | --- | --- | --- | --- | --- | --- | --- |
|  |  | NUCL | CTRL |  | SEM |  | Breeding centre | Group | Group*breeding centre interaction |
| Total protein (g/dL) |  | 5.75 | 5.75 |  | 0.112 |  | 0.29 | 0.96 | 0.76 |
| Albumin (g/dL) |  | 2.58 | 2.75 |  | 0.076 |  | 0.27 | 0.25 | 0.84 |
| α1-globulin (g/dL) |  | 0.18 | 0.18 |  | 0.007 |  | 0.26 | 0.79 | 0.96 |
| α2-globulin (g/dL) |  | 0.45 | 0.48 |  | 0.013 |  | 0.48 | 0.37 | 0.99 |
| β1 globulin (g/dL) |  | 0.81 | 0.82 |  | 0.019 |  | 0.67 | 0.79 | 0.46 |
| β2-globulin (g/dL) |  | 0.69 | 0.68 |  | 0.021 |  | 0.79 | 0.80 | 0.82 |
| γ-globulin (g/dL) |  | 1.07 | 1.08 |  | 0.042 |  | 0.78 | 0.99 | 0.58 |
| TNF-α (ng/L) |  | 68.1 | 66.8 |  | 2.278 |  | 0.61 | 0.75 | 0.26 |
| IFN-γ (ng/L) |  | 46.0 | 50.4 |  | 2.355 |  | 0.90 | 0.39 | 0.28 |
| IL-6 (ng/L) |  | 5.22 | 5.97 |  | 0.401 |  | 0.23 | 0.39 | 0.70 |
| IL-12 (pg/mL) |  | 16.1 | 13.8 |  | 2.389 |  | 0.57 | 0.67 | 0.95 |
| Calprotectin (ng/mL) |  | 53.0 | 54.0 |  | 0.985 |  | 0.19 | 0.58 | 0.16 |
