# Supplementary figures and images for: Dietary supplementation of new-born foals with free nucleotides positively affects neonatal diarrhoea management
Source: Ir Vet J. 2025 Mar 1;78:7. doi: 10.1186/s13620-025-00294-3 (PMC11871744; doi:10.1186/s13620-025-00294-3)

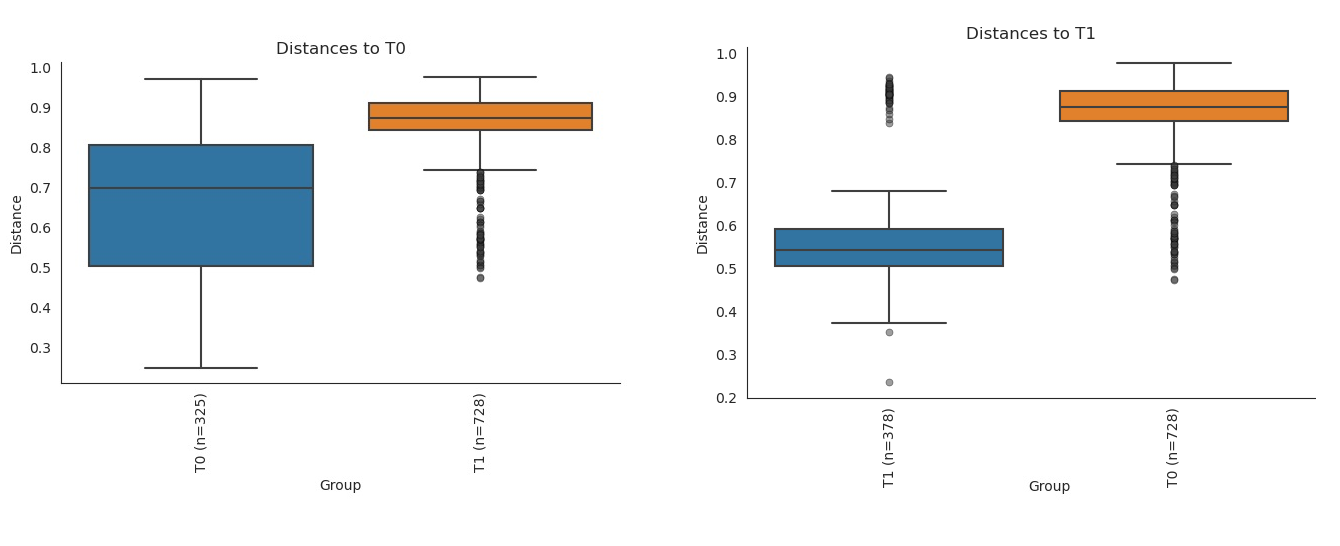

Supplement: Supplementary file 3 — Supplementary Material 3 [file 13620_2025_294_MOESM3_ESM.png]
